# Pathogen exposure influences immune parameters around weaning in pigs reared in commercial farms

**DOI:** 10.1186/s12865-022-00534-z

**Published:** 2022-12-10

**Authors:** Julie Hervé, Karine Haurogné, Arnaud Buchet, Elodie Bacou, Grégoire Mignot, Marie Allard, Mily Leblanc-Maridor, Solenn Gavaud, Anne Lehébel, Elena Terenina, Pierre Mormède, Elodie Merlot, Catherine Belloc, Jean-Marie Bach, Blandine Lieubeau

**Affiliations:** 1 Oniris, INRAE, IECM, Nantes, France; 2 Institut Agro, INRAE, PEGASE, St Gilles, France; 3 Oniris, INRAE, BIOEPAR, Nantes, France; 4 Cooperl Innovation, Pôle Sciences Animales, Lamballe, France; 5 Université de Toulouse, ENVT, INRAE, GenPhySE, Castanet-Tolosan, France

**Keywords:** Immunity, Pig, Pathogen exposure, Weaning, On-field study

## Abstract

**Background:**

Multiple antigenic stimulations are crucial to immune system training during early post-natal life. These stimulations can be either due to commensals, which accounts for the acquisition and maintenance of tolerance, or to pathogens, which triggers immunity. In pig, only few works previously explored the influence of natural exposition to pathogens upon immune competence. We propose herein the results of a multicentric, field study, conducted on 265 piglets exposed to contrasted pathogen levels in their living environment. Piglets were housed in 15 different commercial farms, sorted in two groups, low (HS^LOW^)- and high (HS^HIGH^)-health status farms, depending on their recurrent exposition to five common swine pathogens.

**Results:**

Using animal-based measures, we compared the immune competence and growth performances of HS^LOW^ and HS^HIGH^ pigs around weaning. As expected, we observed a rise in the number of circulating leucocytes with age, which affected different cell populations. Monocyte, antigen-experienced and cytotoxic lymphocyte subpopulation counts were higher in piglets reared in HS^LOW^ farms as compared to their HS^HIGH^ homologs. Also, the age-dependent evolution in *γ*δ T cell and neutrophil counts was significantly affected by the health status. With age, circulating IFNα level decreased and IgM level increased while being greater in HS^LOW^ piglets at any time. After weaning, LPS-stimulated blood cells derived from HS^LOW^ piglets were more prone to secrete IL-8 than those derived from HS^HIGH^ pigs did. Monocytes and granulocytes issued from HS^LOW^ pigs also exhibited comparable phagocytosis capacity. Altogether our data emphasize the more robust immunophenotype of HS^LOW^ piglets. Finally, piglets raised under higher pathogen pressure grew less than HS^HIGH^ piglets did and exhibited a different metabolic profile. The higher cost of the immune responses associated with the low farm health status may account for lower HS^LOW^ piglet performances.

**Conclusions:**

Altogether, our data, obtained in field conditions, provide evidence that early exposure to pathogens shapes the immune competence of piglets. They also document the negative impact of an overstimulation of the immune system on piglets’ growth.

**Supplementary Information:**

The online version contains supplementary material available at 10.1186/s12865-022-00534-z.

## Background

If the development of the immune system is initiated during the foetal life, it is still largely incomplete at birth. This accounts for the increased susceptibility of neonates to clinically relevant infections [1]. During the neonatal period, the microbial colonisation of mucosal tissues plays a key role in the shaping of immunity, especially facilitating tolerance to commensal micro-organisms [2]. Post-natal maturation of the immune system also requires training through multiple non-commensal antigenic stimulations. Thus, the overall immune competence of an individual depends on the diversity of antigens he had been confronted to, particularly early in life. Early pathogen exposure obviously influences the immune landscape of an individual, through multiple mechanisms. For example, it can imprint the human T cell repertoire with potential consequences for pathogen defences [3].

Classical animal models are poorly relevant to elucidate the training effect of pathogen exposure on individuals. It is obviously the case for mice housed in standardised, specific pathogen-free (SPF) conditions, which are immunologically inexperienced. To overcome this limitation, Beura et al*.* worked on mice trapped from free-living feral populations [4]. They demonstrated that these “dirty” mice exhibited an experienced immune phenotype, close to the human one, in contrast with the one of age-matched SPF mice. Pig exhibits strong anatomical, physiological and immunological similarities with human, while being housed in diverse environments [5]. As such, it appears as a valuable model to address this question.

In pig, to our knowledge, only few works purposefully explored how natural exposition to pathogens triggers heterologous immunity. These studies were essentially conducted on adult pigs, reared in experimental facilities. For example, Clapperton et al*.* analysed the effects of the farm health status, defined by the presence or not of specific pathogens, on some immune parameters. In pigs older than 3 months, they reported no major effect on blood monocyte and lymphocyte sub-populations counts [6]. Other studies compared the effects of hygiene conditions on immune parameters, using “dirty” *vs* “clean” environments, to mirror different levels of antigenic stimulation. For example, van der Meer et al*.* evidenced a poor effect of hygiene conditions on white blood cell counts during the fattening period [7]. In other studies, bad hygiene conditions were rather shown to induce greater circulating neutrophil numbers in pigs [8, 9].

In commercial farms, pigs are naturally exposed to variable pathogen pressures. As a consequence, field studies are particularly relevant to document the link between pathogen exposure and immune competence. Along pig’s life, weaning is the most critical step. Piglets are abruptly separated from their mother and mixed with non-littermates, while being exposed to a novel biotic environment. This stressful transition, usually characterised by a decreased food intake and a subsequent growth slowdown, can also triggers long-term consequences for pigs’ health [10].

In this context, we report herein the results of a field study conducted on 265 pigs naturally exposed to contrasted pathogen levels. For this purpose, we selected 15 commercial farms sorted in two groups, low (HS^LOW^) and high (HS^HIGH^) health status farms, depending on their recurrent exposition to five common swine pathogens. Using animal-based measures, we compared the immune competence and growth performances of HS^LOW^ and HS^HIGH^ pigs around weaning.

## Results

We followed the evolution of 17 immune and 4 metabolic parameters in 142 HS^LOW^ and 123 HS^HIGH^ piglets, sampled and weighed two days before and seven days after weaning. Individual raw data are available in Additional file 6 while a summary is presented in Table 1.

**Table 1:** Raw data of immune markers, metabolic parameters and performances in HS^LOW^ and HS^HIGH^ piglets before (t0) and after (t1) weaning

Parameters	HS^LOW^ piglets			HS^HIGH^ piglets		
*Leucocyte counts (10*^*6*^*/ml)*						
Total	t0	139	13.41 [11.15–15.81]	t0	123	11.74 [9.14–13.93]
	t1	141	19.10 [15.25–23.47]	t1	122	16.51 [14.03–19.72]
Neutrophils	t0	139	5.12 [4.07–6.60]	t0	123	4.83 [3.77–6.72]
	t1	141	6.80 [5.05–9.80]	t1	122	6.05 [4.49–8.05]
Lymphocytes	t0	139	7.16 [5.81–8.89]	t0	123	5.69 [4.66–7.00]
	t1	141	9.87 [8.22–12.17]	t1	122	9.04 [7.67–11.0]
Monocytes	t0	139	0.59 [0.41–0.77]	t0	123	0.38 [0.24–0.57]
	t1	141	1.03 [0.77–1.36]	t1	122	0.83 [0.60–1.17]
Eosinophils	t0	139	0.02 [0.01–0.04]	t0	123	0.01 [0–0.03]
	t1	141	0.14 [0.08–0.21]	t1	122	0.12 [0.08–0.18]
*Lymphocyte sub-population counts (10*^*6*^*/ml)*						
B lymphocytes	t0	139	1.06 [0.71–1.36]	t0	121	0.79 [0.60–1.17]
	t1	141	1.38 [1.01–2.02]	t1	122	1.39 [1.06–1.78]
*γ*δ T lymphocytes	t0	122	1.06 [0.80–1.49]	t0	120	0.98 [0.69–1.30]
	t1	138	1.72 [1.32–2.49]	t1	102	2.15 [1.62–2.64]
Naive Th lymphocytes	t0	138	1.40 [1.06–1.76]	t0	120	1.28 [1.01–1.56]
	t1	140	1.82 [1.45–2.30]	t1	120	1.56 [1.24–1.92]
Experienced-Th cells	t0	138	0.39 [0.27–0.53]	t0	120	0.27 [0.21–0.38]
	t1	140	0.63 [0.45–0.88]	t1	120	0.42 [0.32–0.56]
Cytotoxic T cells	t0	139	0.89 [0.60–1.51]	t0	120	0.55 [0.42–0.80]
	t1	141	1.38 [0.99–1.86]	t1	120	0.91 [0.62–1.35]
*Circulating markers*						
IgM (mg/ml)	t0	142	0.76 [0.59–1.02]	t0	123	0.57 [0.46–0.78]
	t1	141	1.30 [0.98–1.74]	t1	122	1.03 [0.78–1.39]
IFNα (U/ml)	t0	85	57.4 [16.1–135.8]	t0	93	32.9 [14.8–119.4]
	t1	85	32.1 [15.1–74.4]	t1	96	25.4 [12.1–92.2]
*Phagocytic capacity (%)*						
Mononuclear cells	t1	142	20 [17.6–24.8]	t1	122	20.2 [16.1–24.6]
Polynuclear cells	t1	142	77.5 [69.2–82.8]	t1	122	76.9 [68.1–83.3]
*Whole Blood Assay (pg/ml)*						
IL-8 secretion	t1	142	142 [62–232]	t1	122	76 [62–123]
IL-10 secretion	t1	123	168 [125–24]	t1	107	129 [94–175]
TNFα secretion	t1	142	68 [25–173]	t1	122	41 [20–78]
*Blood metabolites*						
Glucose (g/l)	t1	124	1.03 [0.95–1.13]	t1	122	1.10 [1.01–1.23]
FFA (mg/l)	t1	138	143.6 [95.7–210.1]	t1	122	191.5 [130.3–281.1]
Creatinine (mg/l)	t1	142	11.69 [10.84–12.76]	t1	122	11.07 [10.10–12.2]
Urea (mg/l)	t1	142	109.1 [67.1–183.8]	t1	122	75.2 [52.1–115.3]
*Performances*						
Weight (kg)	t0	142	7.8 [6.9–8.8]	t0	123	8.5 [7.7–9.5]
	t1	142	9.1 [8.3–10.3]	t1	123	10.3 [9.1–11.6]

For each, the number of samples, median [and interquartile ranges] are reported

### White blood cell counts

**Fig. 1:**
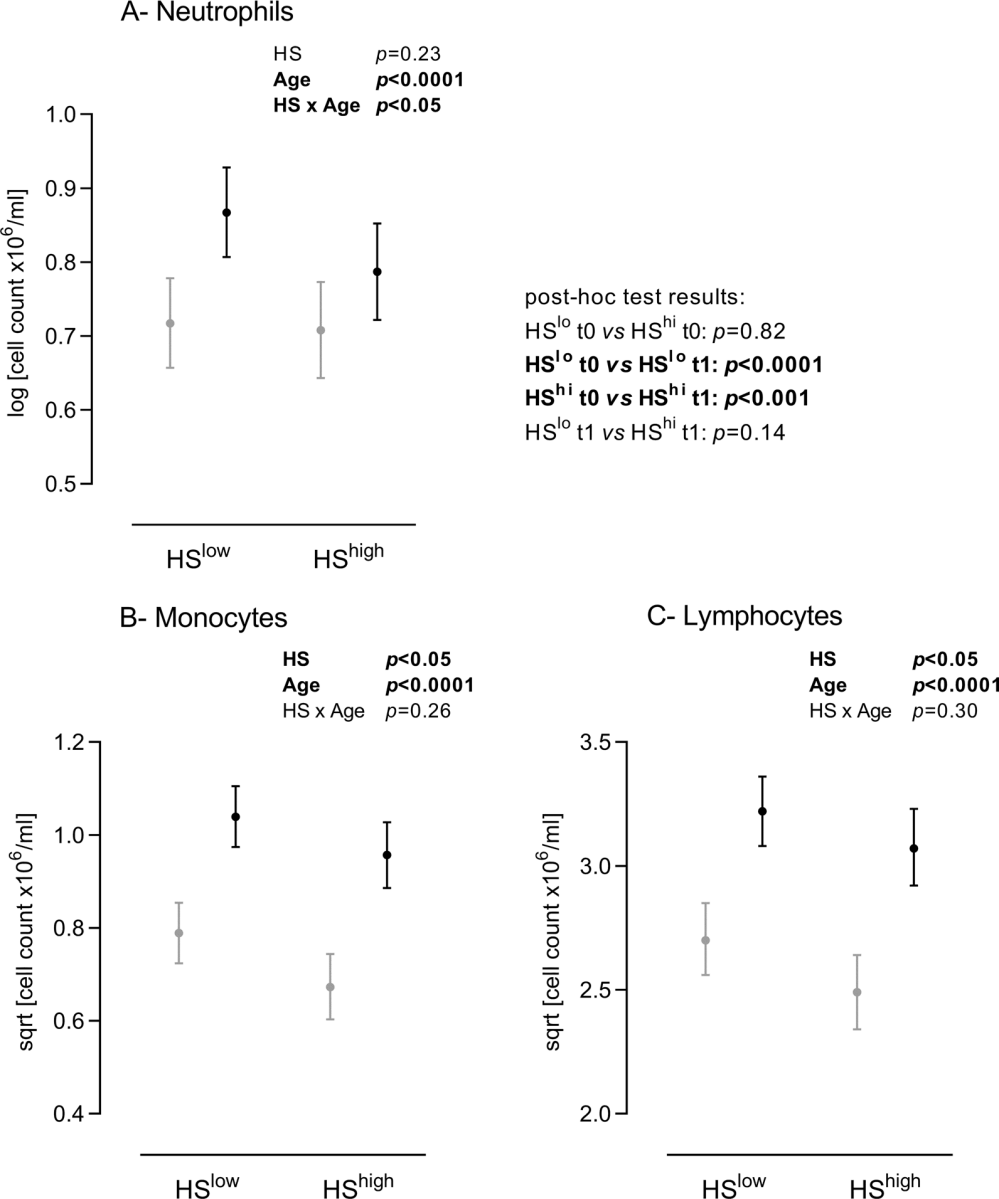
White blood cell counts in HS^LOW^ versus HS^HIGH^ piglets, before (grey) and after (black) weaning. Neutrophil (**A**), monocyte (**B**) and lymphocyte (**C**) numbers were obtained from the hematology analyser. Emmeans with lower and upper confidence intervals’ limits are shown on graphs. Insets, results of the linear mixed-effects model analyses

Neutrophil counts were not different between HS^LOW^ and HS^HIGH^ piglets both before (*p* = 0.82) and after (*p* = 0.14) weaning (Fig. 1a). They increased with age (*p* < 0.0001) but to a larger extent in piglets reared under high pathogen pressure (HS × Age, *p* < 0.05). Monocyte (Fig. 1b) and lymphocyte (Fig. 1c) numbers were affected by both the farm health status (*p* < 0.05) and the age (*p* < 0.0001). At any time, both were significantly greater in piglets reared in the worst environmental conditions. The age-dependent increase in monocyte and lymphocyte counts was not affected by pathogen exposure (HS × Age, *p* = 0.26 and 0.30 respectively). Eosinophils were undetectable or present in very low numbers (detection threshold 10^4^ cells/ml) in piglets before weaning under any condition. Their count dramatically increased after weaning, reaching comparable levels in both groups (Additional file 1). Of note, basophil counts were undetectable-to-low under any condition (data not shown).

### Lymphocyte subpopulations analyses

We next assessed whether the different lymphocyte subpopulations behaved as the whole lymphocyte population did. The gating strategies used for T and B cells’ analyses are described in Additional files 2 and 3 respectively. For Ag-experienced Th (A) and cytotoxic T (B) lymphocytes, cell counts were affected by both the farm health status (*p* < 0.01) and the age (*p* < 0.0001), without interaction (Additional file 4). Cell numbers increased with age in any of the environmental conditions and were higher in HS^LOW^ piglets at any time. After weaning, the proportion of antigen-experienced cells among CD4^+^ T lymphocytes was 25.8% in piglets reared under low farm health status versus 21.7% in HS^HIGH^ pigs.

**Fig. 2:**
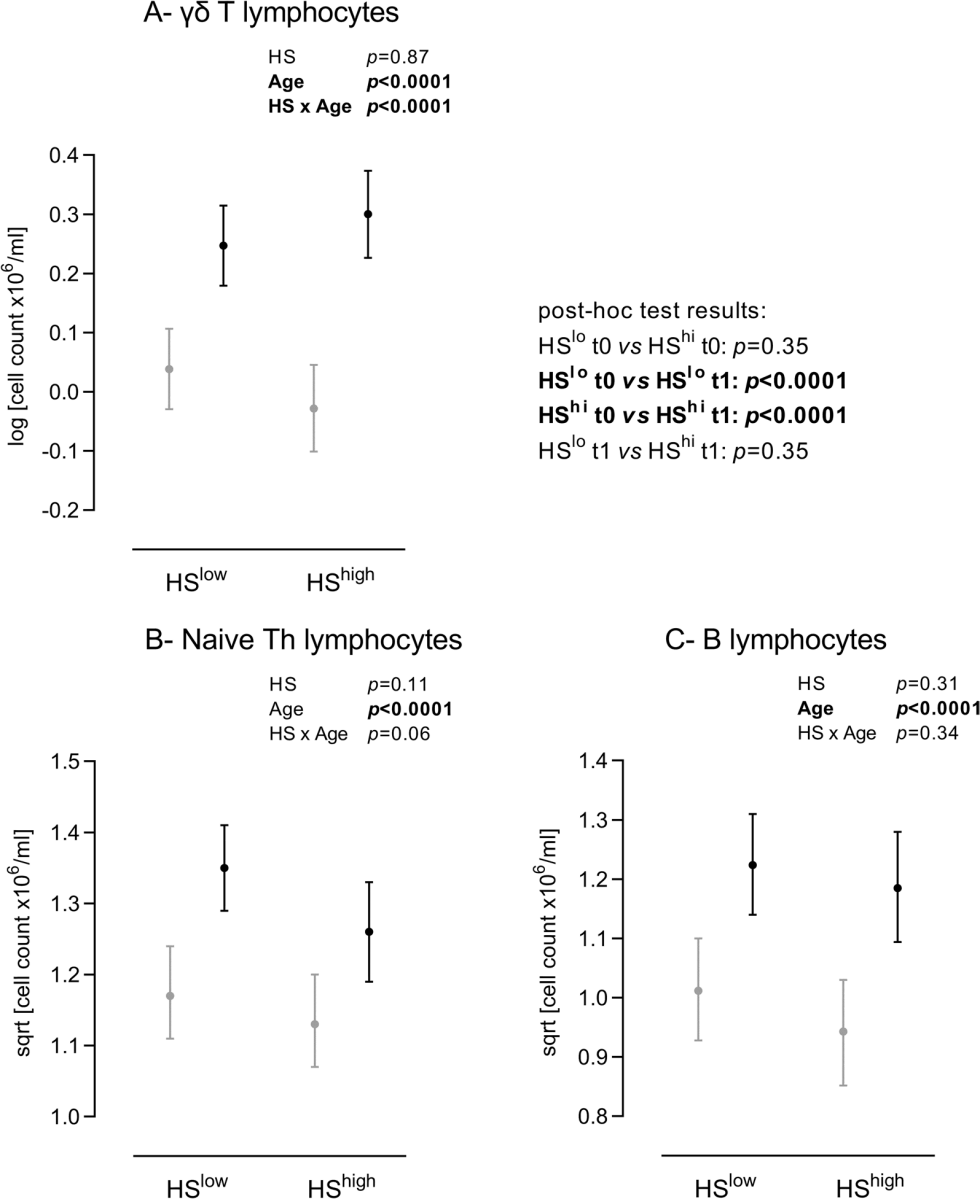
*γ*δ T, naive CD4^+^ T and B lymphocyte counts in HS^LOW^ versus HS^HIGH^ piglets before (grey) and after (black) weaning.* y*δ T lymphocytes (**A** CD3^+^ CD4^−^ CD8^−/med^), naive CD4^+^ Th lymphocytes (**B** CD3^+^ CD4^+^) and B lymphocytes (**C** CD3^−^ CD21^+^) were discriminated using flow cytometry and their numbers calculated. Emmeans with lower and upper confidence intervals’ limits are shown on graphs. Insets, results of the linear mixed-effects model analyses

As shown in Fig. 2, three lymphocyte subpopulations, CD8α^−^ *γ*δ T (A), naive Th (B) and B (C) lymphocytes were affected by the age (*p* < 0.0001) but not by the farm health status (*p* = 0.87, 0.11 and 0.31 respectively). Interestingly, the age-dependent increase in the CD8α^−^ *γ*δ T lymphocyte count was greater in HS^HIGH^ piglets which were reared under low pathogen pressure (HS × Age, *p* < 0.0001).

### Plasma levels of IgM and IFNα

**Fig. 3:**
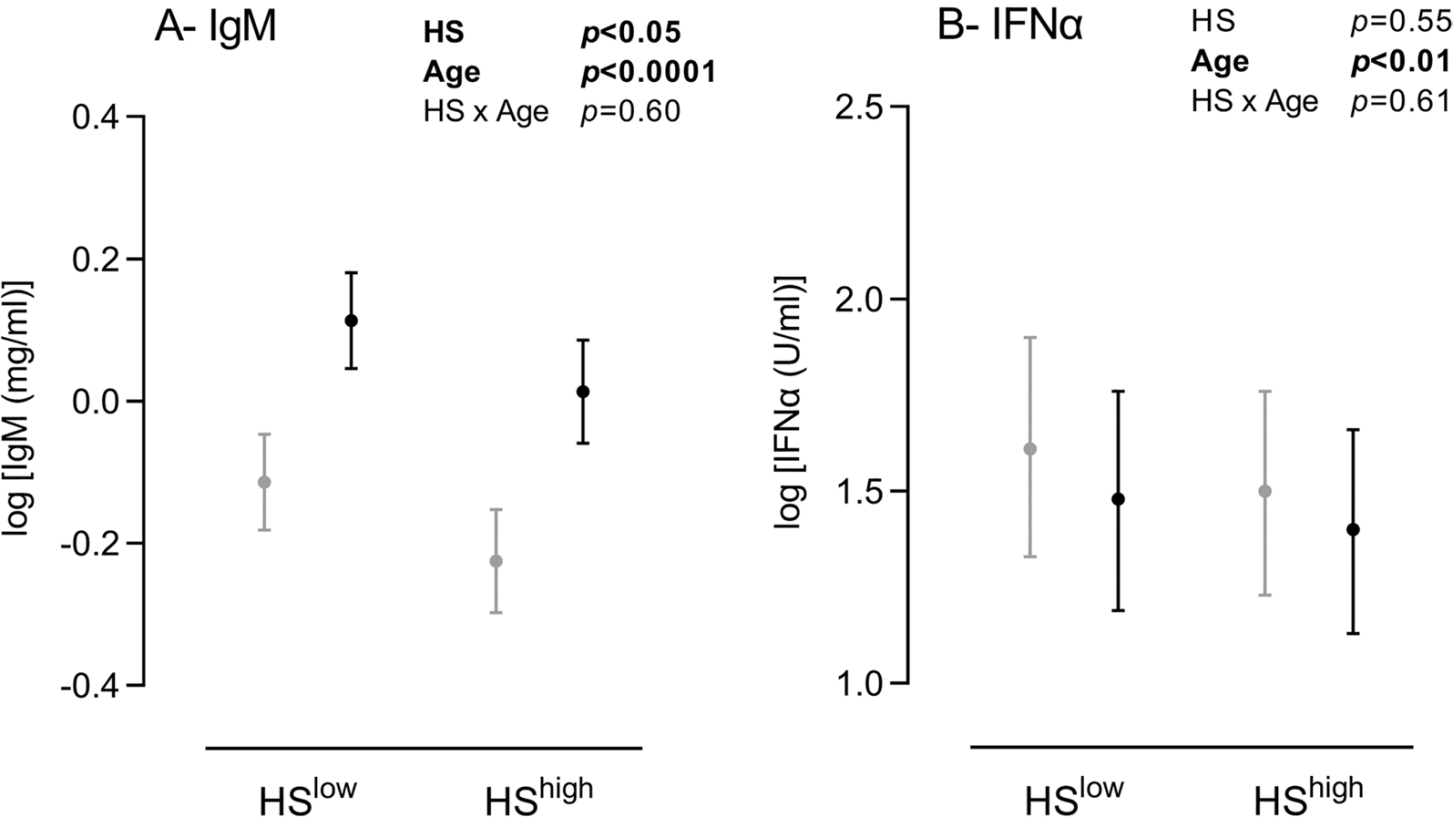
Plasmatic IgM and IFNα in HS^LOW^ versus HS^HIGH^ piglets before (grey) and after (black) weaning. IgM (**A**) and IFNα (**B**) levels were measured in blood samples. Emmeans with lower and upper confidence intervals’ limits are shown on graphs. Insets, results of the linear mixed-effects model analyses

Both the farm health status (*p* < 0.05) and the age (*p* < 0.0001) affected circulating IgM concentration, without interaction (HS × Age, *p* = 0.60) (Fig. 3a). Indeed, piglets reared under HS^LOW^ conditions displayed greater IgM levels, both before and after weaning. With age, we observed a significant rise in IgM concentration (*p* < 0.0001). We also evidenced a decrease in IFNα concentration with age (Fig. 3b; *p* < 0.01), independently of pathogen pressure.

### Assessment of immune competence after weaning

**Fig. 4:**
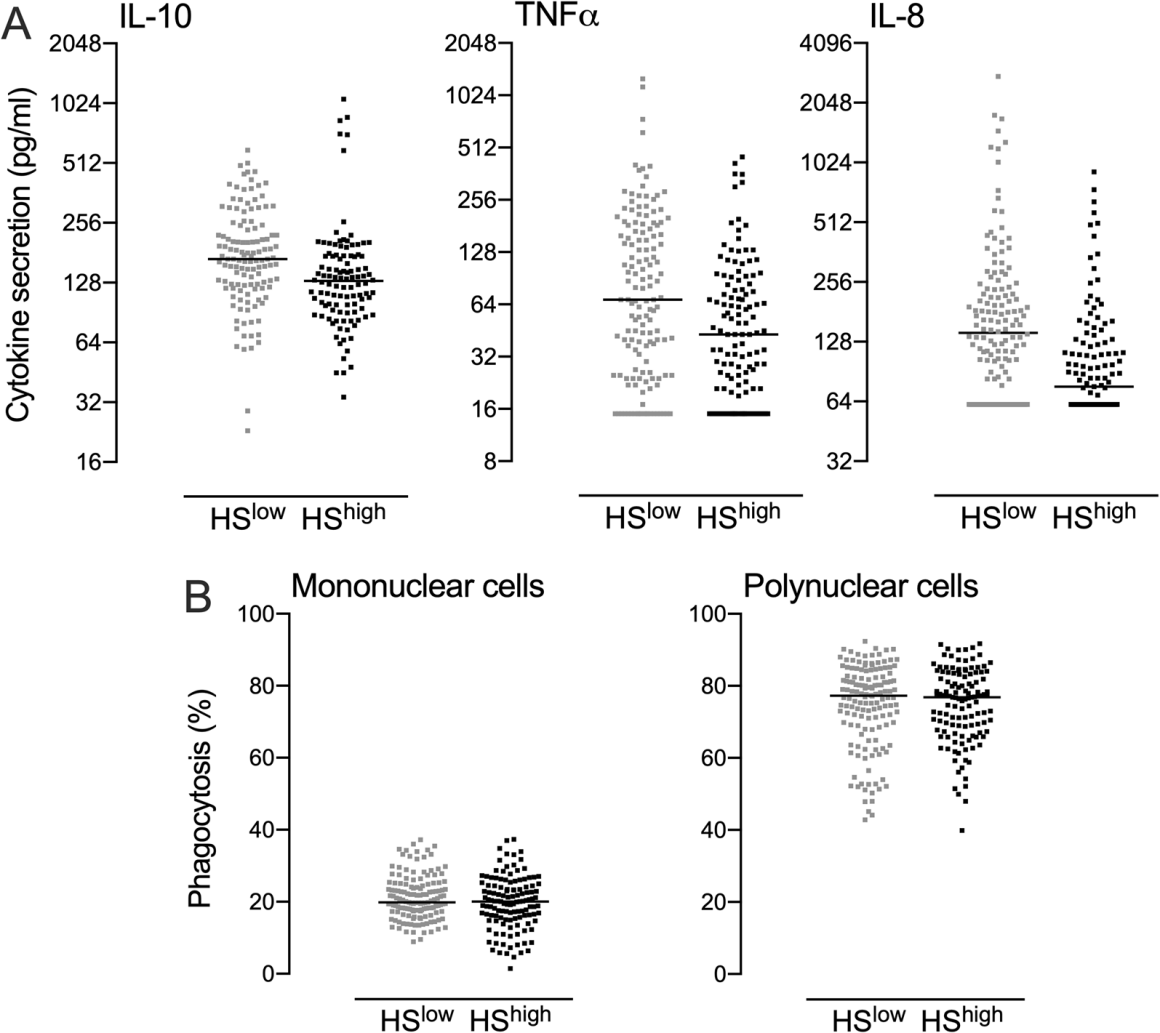
Cytokine secretion and phagocytic capacity of leucocytes issued from HS^LOW^ versus HS^HIGH^ piglets after weaning. **A** Secretion of IL-10, TNFα and IL-8 was measured in response to the ex vivo exposure of blood cells to LPS in WBA. **B** Fluorescent bacteria phagocytosis by blood cells was analysed by flow cytometry in HS^LOW^ versus HS^HIGH^ piglets. Medians are shown on graphs

To further evaluate the ability of piglets to cope with pathogen challenge, functional tests were performed seven days after weaning and raw data are shown on Fig. 4.

We first measured the ability of blood cells to secrete IL-10, TNFα and IL-8 in response to an ex vivo stimulation by LPS (Fig. 4a)*.* IL-10 secretion by blood cells was not significantly modulated by the health status of piglets (*p* = 0.5). Since TNFα and IL-8 were not detectable in 49 and 94 upon 264 samples respectively, we analysed these parameters as qualitative variables. TNFα detection was not modulated by the health status (*p* = 0.35). In contrast, blood cells derived from HS^LOW^ piglets were more likely to secrete detectable levels of IL-8 than HS^HIGH^ piglets did (Odd ratio = 0.41 [0.23–0.67], *p* < 0.001). Finally, the phagocytosis ability of blood neutrophils and monocytes was not affected by the farm health status (Fig. 4b).

### Plasma metabolite concentrations after weaning

We measured circulating glucose, free fatty acid, urea and creatinine levels in fasted piglets one week after weaning and synthesised them using a Principal Component Analysis (PCA) (Additional file 5). The first three components explained more than 80% of the total inertia and each contributed to more than 20% of the total variance. The first dimension was related to “alternative energy supply” while the second one represented “major energy stores”, finally, the third dimension appeared more integrative with equivalent contributions of glucose, free-fatty acids and urea. Dimensions 1 and 2 were not modulated by the farm health status. In contrast, the third dimension was significantly affected by the health status (*p* < 0.001) with the metabolic profile of HS^LOW^ piglets (emmeans = − 0.37 [− 0.70 to − 0.03]) shifted towards urea instead of glucose and free-fatty acids such as the one of HS^HIGH^ piglets (emmeans = 0.43 [0.07–0.79]).

### Individual growth performances and clinical signs

Finally, piglets were weighed at the two time points (see Table 1 for raw data). Before weaning, the weight of HS^LOW^ and HS^HIGH^ piglets was not different with estimated marginal means of 7.82 kg [7.31–8.32] for HS^LOW^ piglets, and 8.20 kg [7.66–8.75] for HS^HIGH^ piglets (*p* = 0.22). In contrast, along the period we studied, HS^LOW^ piglets grew significantly less than HS^HIGH^ piglets did, with estimated marginal means of 158 [126–190] and 219 [185–253] g/day respectively (*p* < 0.05).

Along the period we studied, we detected no major health problems in any of the groups. After weaning, at day 35, only fifteen individuals upon 265 exhibited diarrhoea and the diarrhoeic piglets were equally distributed between HS^LOW^ and HS^HIGH^ farms (Additional file 6).

## Discussion

In this field study, we demonstrated the impact of pathogen exposure on the immune system maturation and growth performances of piglets around weaning. For this purpose, we took advantage of the natural exposure of piglets to common swine pathogens in commercial farms. Using individual blood indicators, we compared the immune competence and the growth of male piglets two days before and one week after weaning.

As already described, we observed a rise in the number of circulating leucocytes with age, which affected different cell populations. At weaning, the immune system is still developing, circulating cell numbers reaching stable levels after the age of 10 weeks [11]. The neutrophil count increased less with age than the lymphocyte count did, which resulted in a decrease in the neutrophil-to-lymphocyte ratio (data not shown). According to Stull et al*.*, this decrease persists until the acquisition of the immune competence [12].

As we expected, the farm health status significantly affected several immune parameters. Plasma IgM levels were greater in piglets grown under HS^LOW^ conditions compared to HS^HIGH^ piglets. After birth, piglets acquire maternal immunoglobulins, mainly IgG from colostrum, before producing their own, starting with IgM [13]. Thus, the greater IgM levels appear as the consequence of a stronger antigenic stimulation due to the higher microbial pressure in HS^LOW^ farms.

To characterise the effects of the farm health status on lymphocytes, we focused on subpopulations that were robustly quantified by flow cytometry on blood samples. Indeed, while it could have been interesting to quantify regulatory T cells and Th17 cells in our samples, intracellular staining is poorly adapted to whole-blood samples. We observed significantly greater numbers of antigen-experienced and cytotoxic T lymphocytes in piglets raised in HS^LOW^ farms. In swine, CD3^+^* γ*δ T lymphocytes can represent up to 50% of total circulating lymphocytes [14]. This lymphocyte subtype is crucial to young animals, before the complete maturation of their immune system [15]. In particular, the CD8α^−^ fraction of *γ*δ T cells was shown to dominate within the peripheral blood lymphocyte compartment in pigs [16]. In this context, the evolution of CD8α^−^ *γ*δ T cell counts with age in piglets raised under low or high pathogen pressure is particularly interesting. Indeed, the increase in CD8α^−^ *γ*δ T cell counts around weaning was lower in HS^LOW^ piglets raised under high pathogen pressure. In contrast, in these piglets, the age-dependent rise in the neutrophil count was more pronounced. Altogether, these characteristics may be considered as hallmarks of a more mature immunophenotype in HS^LOW^ piglets.

The concept of trained immunity was initially described in plants and invertebrates. It refers to the ability of innate immune cells to secrete higher amounts of pro-inflammatory cytokines when exposed to a second, unrelated, damage-associated molecular pattern [17]. This innate immune memory was demonstrated to be crucial for juveniles that still lack efficient adaptative immunity [18]. After weaning, while we did not evidence any difference in LPS-induced IL-10 and TNFα secretion, we observed that leucocytes derived from HS^LOW^ piglets were more prone to secrete the pro-inflammatory cytokine IL-8. Although we cannot exclude a bias related to the greater monocyte count evidenced in HS^LOW^ pigs, it might reveal different trained immunity patterns. Indeed, the stronger capacity of their leucocytes to respond to LPS could confer to HS^LOW^ pigs a better ability to face Gram-negative bacterial infections as previously suggested for humans [19]. HS^LOW^ pigs also exhibited greater numbers of monocytes with comparable phagocytosis capacity, emphasising their more robust immunophenotype.

We also confirmed herein the extra cost of the immune training through antigenic stimulation in HS^LOW^ pigs. It is well known that individuals have to balance the fitness benefits of a robust immune response against the costs of drawing resources [20]. In agreement with this concept, piglets born and reared in HS^HIGH^ farms grew better than their homologs reared under low health status. Since we did not measure the feed consumption of piglets, we do not know whether HS^LOW^ piglets ate less than HS^HIGH^ piglets did. Nevertheless, it has already been documented that poor sanitary conditions after weaning can deteriorate growth performances despite a limited negative effect on feed intake [21]. We presume that the decreased performances of HS^LOW^ piglets were likely due to the nutritional cost of adaptation to the higher pathogen pressure as previously described [22] and suggested by the different metabolic profiles of the HS^LOW^ and HS^HIGH^ piglets one week after weaning.

One of the limitations of this study is that it was performed on males only, with the aim to reduce heterogeneity due to the well-known influences of the gender on immune responses [23]. Early in life, in humans, circulating immune cell counts are generally greater in males than females. In young pigs also, some immune parameters were shown to differ between males and females [24]. In this context, part of the differences we observed might not have been seen in females. The season might constitute another bias of this study. Indeed, piglets were either born in winter or early spring, which may have influenced the circulation of pathogens. However, samplings in HS^LOW^ farms were interspersed by experiments in HS^HIGH^ farms. Finally, in this multicentric, on-field study, although we could not control the whole environmental conditions, we selected the farms as relatively homogenous in terms of genetics and batch management production system. In order to reduce the bias related to other parameters such as feed or ambient conditions (air quality including relative humidity or dust and ammonia concentration…), we took into account the farm effect in the statistical models we used.

In this study, the two defined health statuses did not represent highly contrasted situations since they were representative of field conditions found in commercial farms. As a consequence, at any time point, the large majority of piglets was healthy. After weaning, only few piglets expressed digestive symptoms, which is in accordance with the farmers’ health recordings made between day 26 and day 48 in each post-weaning room. Indeed, as previously reported, the total number of days with recorded diarrhoea was 26 in the HS^LOW^ farms compared to 22 in the HS^HIGH^ farms [25]. Nevertheless, the impact of pathogen exposure on immune parameters was not insignificant which confirms that early exposure to pathogens shapes the immune competence of piglets. Recent findings reported that housing conditions in early life influenced specific antibodies and leucocyte populations in older piglets, suggesting quite long-term effects [26]. Whether the differences we observed in some immune parameters persist or alleviate all along pigs’ life will have to be explored.

## Conclusions

Our data obtained in field conditions describe the immune and metabolic consequences of variable levels of pathogen exposure on piglets, echoing previous findings connecting pathogen exposure to immune system shaping. It also dovetails with previous literature confirming the nutritional cost associated with the overstimulation of the immune system.

## Methods

### Ethical statement

All experimental procedures were carried out in accordance with the European directive 2010/63/EU for animal experiments. It was submitted to and approved by the Oniris’ ethics committee for veterinary clinical research (decision CERVO-2016-6-V).

### Selection of the farms

This study was conducted in 15 commercial farms in the West of France during a 5 month-period, from January to May. Farms were selected a priori based on the analysis of the annual farm health records made by the attending veterinarians. Recurrent exposure to five major porcine pathogens, namely Influenza virus H1N1, Porcine Reproductive and Respiratory Syndrome virus, *Streptococcus suis*, *Actinobacillus pleuropneumoniae* and *Lawsonia intracellularis*, was used to determine farms’ health status (HS). Farms were further classified as HS^LOW^, when more than 2 pathogens were detected, or conversely HS^HIGH^. Each farm health status was confirmed at the end of the study using the record of the year. The overall characteristics of the farms (batch management system, animals’ genetics, nutrition, post-weaning facilities…) are provided in Additional file 7. Of note, farms were located in the periphery of the city of Nantes to allow rapid processing of the samples (transport time < 2 h).

### Animals, experimental design and sample collection

We studied healthy entire male piglets [Nucleus germ line: Pietrain x (Landrace x Large White)]. Indeed, in the selected farms, castration was no longer practiced. In each farm, 18 individuals, issued from 9 sows (2 piglets/sow) of different parities (representative of the demographic pyramid of the farm), were initially included. We selected middleweight piglets upon visual inspection of the litters. Unless specified in the Table 1, analyses were performed on 142 HS^LOW^ versus 123 HS^HIGH^ piglets (respectively from 8 and 7 farms), due to the exclusion of five deceased piglets equally distributed between both groups.

Blood sampling was performed in the morning, two days before and one week after weaning (that occurred at day 28), when piglets were in average 26 (t0) and 35 (t1) day-old. For the second sampling, piglets had been fasted overnight to allow biochemical analyses (i.e. glycemia, creatininemia, uremia and free-fatty acidemia). Blood was collected from the jugular vein of pigs slightly maintained in a supine position, by experienced staff, in less than 30 s per pig. The total volume of blood collected never exceeded 25 ml per pig. Immediately after blood sampling, piglets were individually weighed using a portable electronic scale. Careful examination of the animal was performed to detect any clinical sign. At t1, rectal temperature was measured and the occurrence of diarrhoea was estimated upon faeces consistency (liquid or not).

### Complete blood cell counts

Complete blood cell counts were determined from EDTA-treated blood samples using the clinical-grade Procyte Dx haematology analyser (IDEXX, Saint-Denis, France). White blood cell parameters included total white blood cell (WBC), neutrophil, eosinophil, lymphocyte and monocyte counts.

### Analyses of lymphocyte subpopulations

Lymphocyte subpopulations were directly identified from EDTA-treated blood samples using two types of whole-blood staining with fluorochrome-conjugated monoclonal antibodies: (1) FITC-conjugated anti-pig CD4 (Acrys Antibodies, Herford, Germany), PE-conjugated anti-pig CD8α (BD Biosciences) and PerCP-conjugated anti-pig CD3 (Bio-Techne, Lille, France) antibodies, or (2) PE-conjugated anti-pig CD21 (Acrys Antibodies) and PerCP-conjugated anti-pig CD3 in the dark for 15 min at room temperature. Erythrocytes were removed using a lysis solution (BD Biosciences, Le Pont de Claix, France) and the DNA marker DRAQ5™ (1 μM final; Biostatus, Leicestershire, UK) was added. Samples were analysed with a FACS Aria flow cytometer (BD Biosciences, Le Pont de Claix, France) and data computed using the FlowJo software (FlowJo, Ashland, USA). Staining allowed the identification of five different lymphocyte subpopulations: B lymphocytes (CD3^−^ CD21^+^), naive CD4^+^ Th cells (CD3^+^ CD4^+^), antigen-experienced Th cells (CD3^+^ CD4^+^ CD8α^+^), CD3^+^ *γ*δ T cells (CD3^+^ CD4^−^ CD8α^−^) and CD8α^hi^ cytotoxic T lymphocytes (CD3^+^ CD4^−^ CD8α^hi^). The cell count for each specific subpopulation was then determined as the product of the percentage of this specific subpopulation in the DRAQ5^+^ FSC/SSC white blood cell gate (at least 15 000 cells per sample analysed and 40 000 for most of them) by the white blood cell count obtained from the Procyte analyser.

### Phagocytosis

Ex vivo phagocytosis was assessed using the Phagotest™ Kit (BD Biosciences). Briefly, 50 µl of heparinised blood were incubated for 10 min at 37 °C with opsonised FITC-labelled *E. coli* bacteria. For each analysis, ice-incubated negative controls were included. Samples were immediately analysed by flow cytometry (20 000 cells/sample analysed) using FSC/SSC dot plot to discriminate granulocytes (FSC^high^) from mononuclear cells (FSC^low^). Results are expressed as percent of phagocytes among mono- and poly-nuclear cells.

### Biochemical analyses

Glycemia, uremia and creatininemia were analysed on heparinised plasma samples collected from fasted piglets, using an automated analyser (Konelab, Thermo Scientific, Courtaboeuf, France) and commercial kits (Thermo Scientific). Free fatty acids levels were determined on EDTA-treated plasma samples with a commercial kit (Wako-Chemicals, Neuss, Deutschland). IgM concentrations were determined by a homemade quantitative sandwich ELISA on heparinised plasma samples. Briefly, serially diluted plasmas were incubated for 1 h in 96-well plates coated with an excess of goat antibody against swine IgM Fc. After washing, a peroxidase-conjugated goat anti-swine IgM Fc was added. Capture and conjugated antibodies were purchased from MyBiosource (Clinisciences, Nanterre, France).

### Plasma and whole blood assay (WBA) cytokine measurements

Porcine IFNα was analysed in heparinised plasma samples using a homemade sandwich ELISA test. The test used K9 anti-IFNα and biotinylated-F17 anti-IFNα antibodies, and recombinant swine IFNα as standard (PBL Assay Science, Piscataway, USA). The intra-assay coefficient of variation (CV) was 3% while inter-assay CV was 18%.

For WBA, heparinised blood samples were diluted five-fold in RPMI 1640 medium and stimulated for 18 h in duplicate using 10 ng/ml *Escherichia coli* O111:B4 LPS (Sigma-Aldrich, St-Quentin-Fallavier, France). Porcine TNFα, IL-8 and IL-10 were then quantified by ELISA with respective detection limits of 15.6, 62.5 and 23 pg/ml (Bio-Techne, Noyal-Chatillon sur Seiche, France).

### Statistical analyses

Statistical analyses were performed using R software (version 4.1.2, R Core Team, 2016) while charts were graphed using Graph Pad Prism (version 9.3.1).

To describe the consequences of the farm health status and/or the age upon immune and endocrine systems in piglets, linear mixed-effects models were performed using the lme4 package [27]. Whenever required, data were initially transformed. We used logarithm (neutrophil, *γ*δ T lymphocyte, Ag-experienced Th cell and cytotoxic lymphocyte counts, blood IgM, IFNα, urea and free fatty acid concentrations) or square root (monocyte, total lymphocyte, naive CD4^+^ T and B lymphocyte counts). The goodness-of-fit of all models (normality and homoscedasticity of residuals and distribution of random effects) was checked by graphic procedures and by Shapiro–Wilk tests.

The main model included, as fixed effects, the farm health status (HS: low and high), the age (Age: t0 and t1), the sow parity (1 versus 2 and above) and the interaction between HS and Age, and, as random effects, the farm (1 to 15) and the individual (Id), as described below.

$$\begin{aligned} & Y_{ijt} = \beta_{0} + Parity_{ijt} \beta_{1} + Score_{ijt} \beta_{2} + Age_{ijt} \beta_{3} + Score_{ijt} Age_{ijt} \beta_{4} + \eta_{ij} + \nu_{j} + \varepsilon_{ijt} \\ & \eta_{ij} \sim Normal\left( {0,\sigma_{\eta }^{2} } \right) \\ & \nu_{j} \sim Normal \left( {0,\sigma_{\nu }^{2} } \right) \\ & \varepsilon_{ijt} \sim Normal\left( {0,\sigma_{\varepsilon }^{2} } \right) \\ \end{aligned}$$

where $Y_{ijt}$ is the quantitative variable of interest of the animal i in the farm j at age t, $Parity_{ijt}$ is the parity in 2 classes (1 vs 2 and +), $Score_{ijt}$ is the Health Status (LOW vs HIGH), $Age_{ijt}$ is the sampling date (t0 vs t1), $Score_{ijt} Age_{ijt}$ is the interaction between age and score, $\eta_{ij}$ is the random effect for animal *i* in the farm *j*. Animal random effect follows a normal distribution with mean 0 and variance $\sigma_{\eta }^{2}$. $\nu_{j}$ is the random effect for farm *j*. Farm random effect follows a normal distribution with mean 0 and variance $\sigma_{\nu }^{2}$. $\varepsilon_{ijt}$ is the residual for the animal i in the farm j at age t which follows a normal distribution with mean 0 and variance $\sigma_{\varepsilon }^{2}$.

We presented only the effects we were interested in, *i.e.* the ones of the HS, the age and their interaction, and all results were adjusted on sow parity. Of note, 7 parameters were also influenced by sow parity, namely neutrophil, lymphocyte and monocyte counts, experienced Th cell numbers, cytotoxic T cell counts and IFNα level. Also, as expected, piglets issued from parity 1 sows exhibited a lower initial weight (7.47 kg [7.03–7.91]) than the other ones (8.55 kg [8.17–8.93]).

When the interaction effect was significant, a Tukey’s Honest Significant Difference post-hoc test was performed to search for relevant differences and results are shown beside the graphs.

For quantitative variables measured only at t1, only the HS and the sow parity were included in the model as fixed effects, and the farm as a random one. For this model, metabolic parameters (glycemia, free-fatty acidaemia, uraemia and creatininemia) were firstly processed using a Principal Component Analysis (PCA) [28] with the FactoMineR package in the R software [29] in order to reduce the number of variables while minimizing information loss.

TNFα and IL-8 variables were analysed as qualitative variables (< or > 15.6 for TNFα and (< or > 62.5 for IL-8) using mixed logistic regression models included the HS, the farm and the sow parity as fixed effects and the farm as a random effect.

To assess the effect of the HS on growth (ADG), the mixed linear model included the health status, the sow parity and the t0 weight as fixed effects, and the farm as a random one.

For most response variables, estimated marginal means (emmeans) and lower and upper limits of the 95% confidence interval [IC95] were calculated using the emmeans package [30] and graphed on charts.

## Supplementary Information

**Additional file 1**. Eosinophil cell counts in HS^LOW^ and HS^HIGH^ piglets after weaning.

**Additional file 2**. Gating strategy for the identification of T lymphocyte sub-populations.

**Additional file 3**. Gating strategy for the identification of B lymphocytes.

**Additional file 4**. Ag-experienced Th (A) and cytotoxic T lymphocyte (B) counts inHS^LOW^ versus HS^HIGH^ piglets before (grey) and after (black) weaning.

**Additional file 5**. Principal Component Analysis on metabolic data.

**Additional file 6**. Individual raw data.

**Additional file 7**. Characterisation of the farms.

## Data Availability

The datasets used and/or analysed during the current study are provided in the supplementary file section.

## Abbreviations

ADG: Average Daily Gain
emmean: Estimated marginal mean
FFA: Free-Fatty Acids
HS: Health status
IC: Confidence Interval
PCA: Principal Component Analysis
SPF: Specific Pathogen Free
WBA: Whole Blood Assay
WBC: White Blood Cell
