# Supplementary material for: Pathogen exposure influences immune parameters around weaning in pigs reared in commercial farms
Source: BMC Immunol. 2022 Dec 10;23:61. doi: 10.1186/s12865-022-00534-z (PMC9737769; doi:10.1186/s12865-022-00534-z)
Supplement: Supplementary file 2 — Additional file 2. Gating strategy for the identification of T lymphocyte sub-populations. [file 12865_2022_534_MOESM2_ESM.pdf]

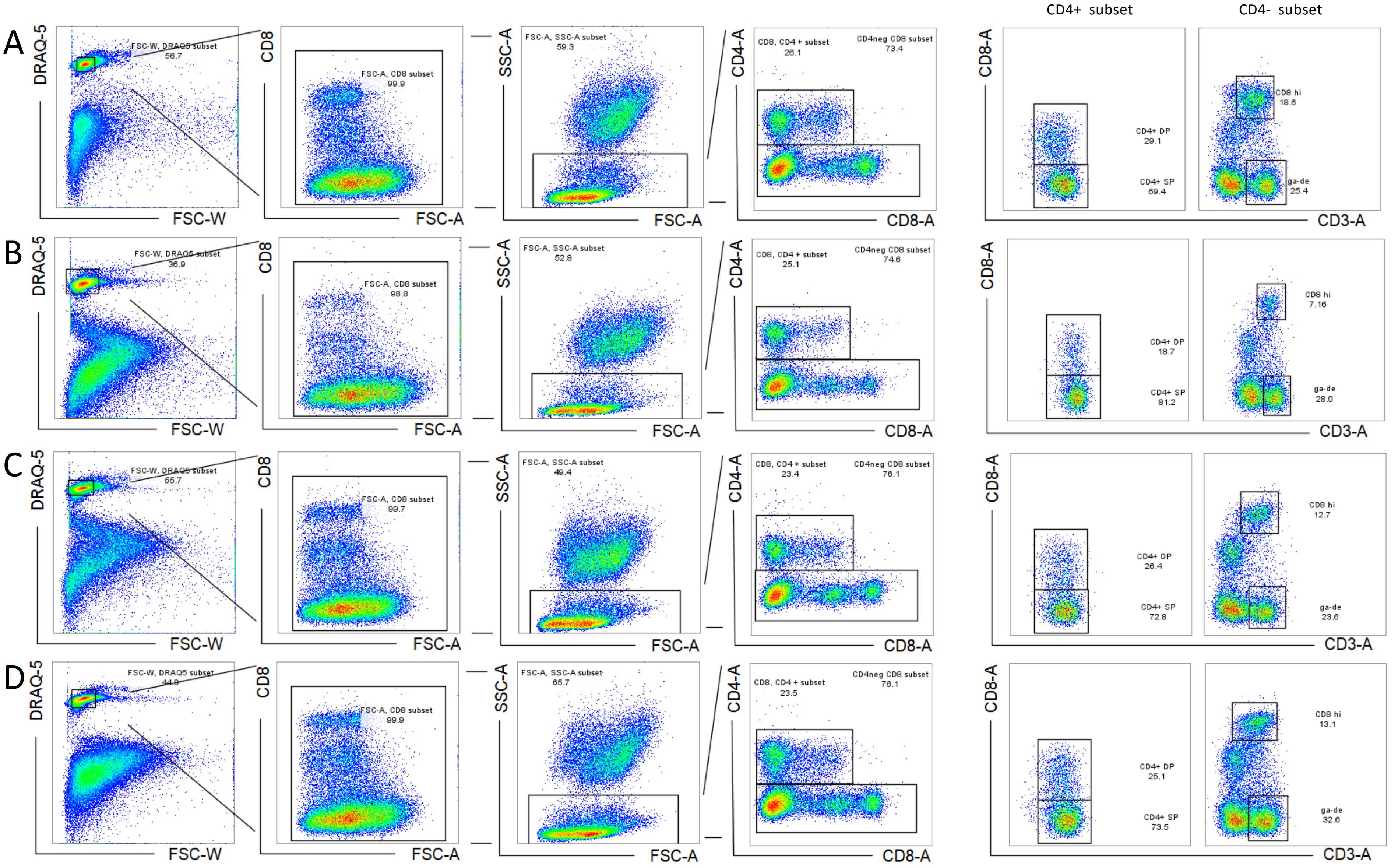

Supplemental Figure 2: Gating strategy for the identification of T lymphocyte sub-populations.
Blood cells were stained with DRAQ-5, and different fluorochrome-conjugated antibodies (FITC-anti CD4, PE-anti CD8 and PerCp anti-CD3) for flow cytometry analyses. Representative samples at t0 (A, B) and t1 (C, D) of HS^{LOW} (A, C) and HS^{HIGH} (B, D) piglets are shown.
