# Supplementary material for: Pathogen exposure influences immune parameters around weaning in pigs reared in commercial farms
Source: BMC Immunol. 2022 Dec 10;23:61. doi: 10.1186/s12865-022-00534-z (PMC9737769; doi:10.1186/s12865-022-00534-z)
Supplement: Supplementary file 3 — Additional file 3. Gating strategy for the identification of B lymphocytes. [file 12865_2022_534_MOESM3_ESM.pdf]

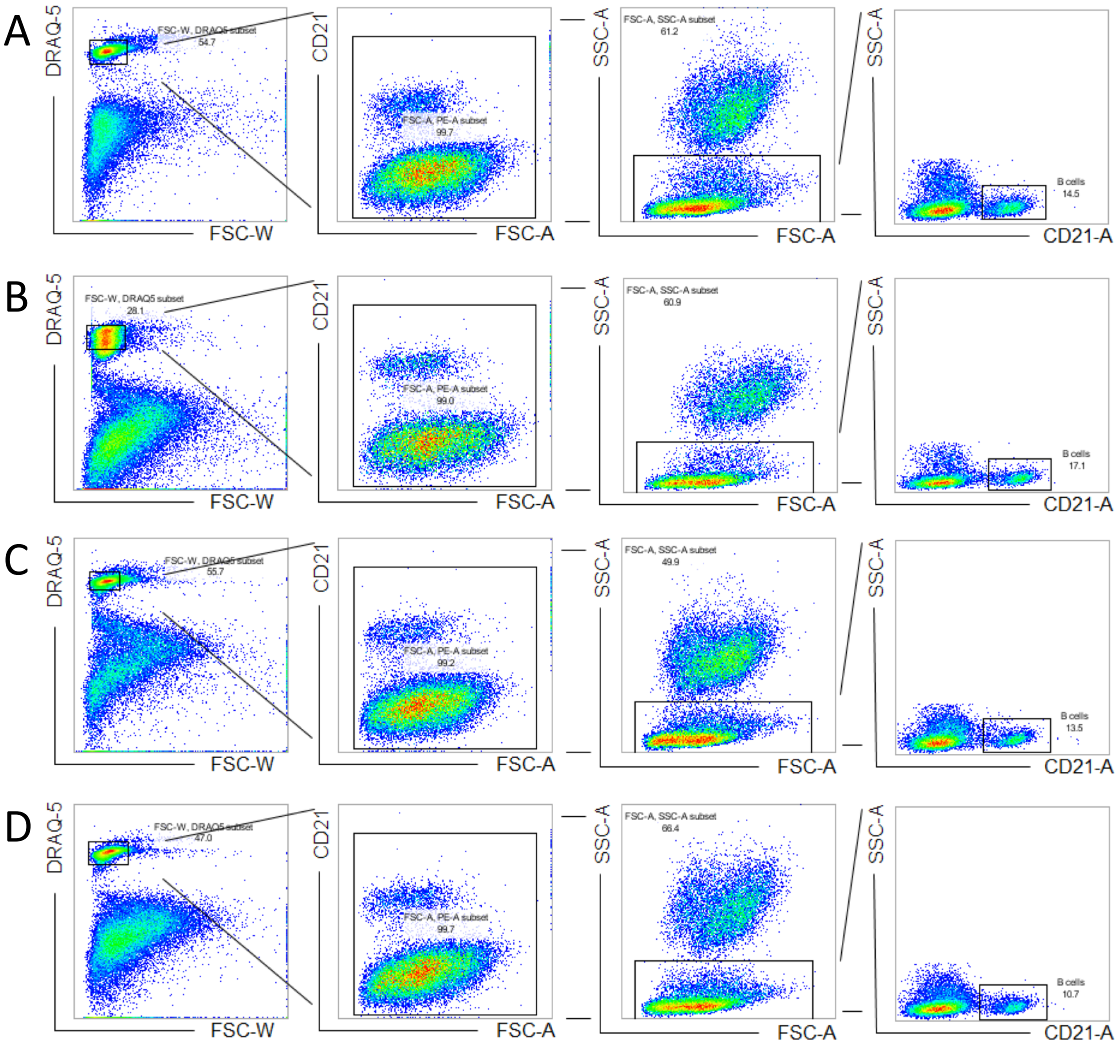

Supplemental Figure 3: Gating strategy for the identification of B lymphocytes.
Blood cells were stained with DRAQ-5 and a CD21-PE-conjugated antibody before flow cytometry analyses.
Representative samples at t0 (A, B) and t1 (C, D) of HS^{LOW} (A, C) and HS^{HIGH} (B, D) piglets are shown.
