# Supplementary figures and images for: Pathogen exposure influences immune parameters around weaning in pigs reared in commercial farms
Source: BMC Immunol. 2022 Dec 10;23:61. doi: 10.1186/s12865-022-00534-z (PMC9737769; doi:10.1186/s12865-022-00534-z)

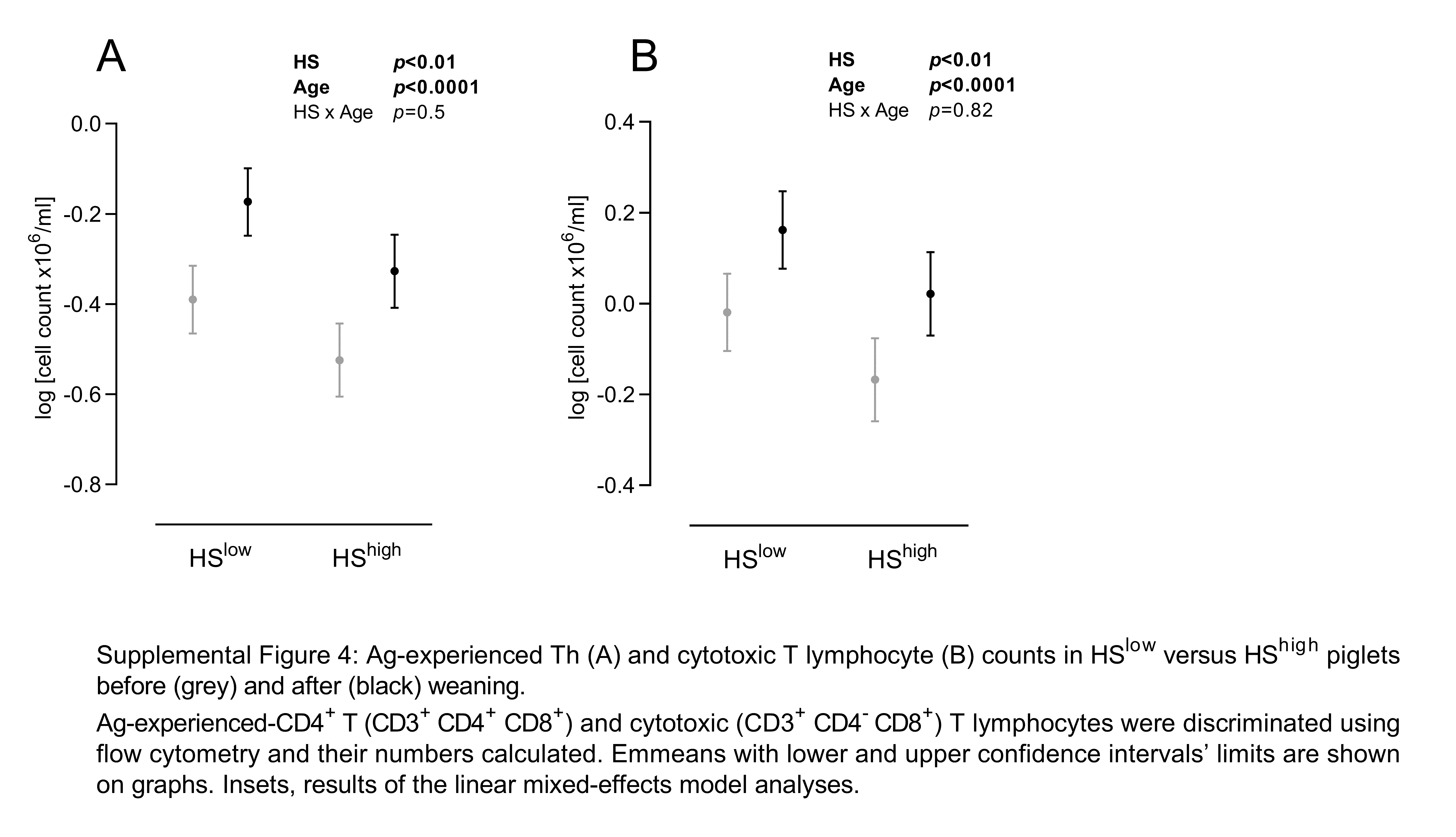

Supplement: Supplementary file 4 — Additional file 4. Ag-experienced Th (A) and cytotoxic T lymphocyte (B) counts inHSLOW versus HSHIGH piglets before (grey) and after (black) weaning. [file 12865_2022_534_MOESM4_ESM.tif]

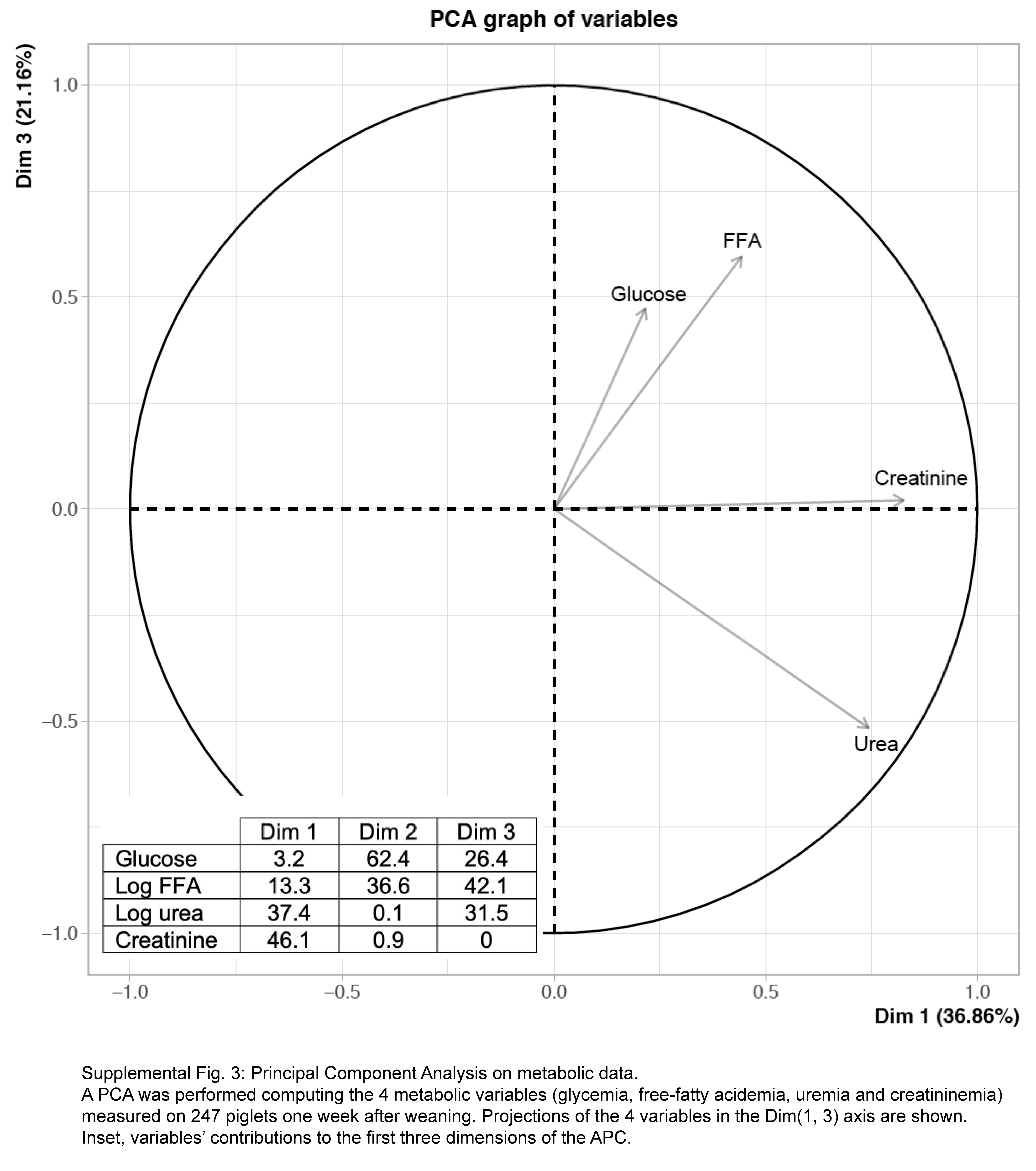

Supplement: Supplementary file 5 — Additional file 5. Principal Component Analysis on metabolic data. [file 12865_2022_534_MOESM5_ESM.tif]
